# From AR to c-Met: Androgen deprivation leads to a signaling pathway switch in prostate cancer cells

**DOI:** 10.3892/ijo.2013.2020

**Published:** 2013-07-18

**Authors:** TIANCHENG LIU, DESIREE E. MENDES, CLIFFORD E. BERKMAN

**Affiliations:** Department of Chemistry, Washington State University, Pullman, WA 99164, USA

**Keywords:** c-Met, androgen receptor, prostate-specific membrane antigen, androgen deprivation, prostate cancer

## Abstract

Elucidating the role of androgen deprivation in the transition from androgen-dependence to independence may enable the development of more specific therapeutic strategies against prostate cancer. Our previous *in vitro* model was employed to further assess the effects of continuous androgen-deprivation on prostate cancer cells (LNCaP) with respect to both androgen receptor (AR) and c-Met expression. The results indicated that long-term androgen deprivation resulted in a signaling pathway switch from AR to c-Met in androgen-sensitive cells, which was confirmed by immunofluorescence imaging and western blot analysis. This signaling pathway switch may be predictive of a more aggressive disease state following androgen deprivation therapy.

## Introduction

Prostate cancer remains the second leading cause of cancer death for men in the United States. According to the National Cancer Institute, it is estimated that there will be 238,590 new cases and 29,720 deaths from prostate cancer in 2013 ( http://www.cancer.gov/cancertopics/types/prostate ). Initially, prostate cancer cells depend upon androgen stimulation for growth and proliferation, and sensitivity to hormone (androgen deprivation) therapy, which effectively blocks the androgen-mediated signaling pathway. Unfortunately most recurrent tumors return within two years with castration-resistant growth and a more aggressive, metastatic phenotype. As of yet, there is no effective treatment for castration-resistant prostate cancer (CRPC) ( 1 , 2 ) .

Recently, several mechanisms have been proposed for the development of castration-resistant prostate cancer ( 3 , 4 ) including mutation, amplification ( 4 , 5 ) , expression alternative-splice variants of the androgen receptor ( 6 ) , or the increase of natural testosterone biosynthesis by cancer cells. These mechanisms suggest that most CRPC cells may depend on androgen receptor function, but are adaptive to low hormone levels. However, clinical evidence and basic research studies support the hypothesis that there are alternative signaling pathways in AR-negative prostate cancer cells or cancer-stem cells ( 7 – 10 ) .

The hepatocyte growth factor/scatter factor (HGF/SF) and its receptor c-Met kinase are key components of the c-Met signaling pathway, which plays a critical role in the regulation of cell growth, cell motility, morphogenesis and angiogenesis during normal development and tissue regeneration ( 11 , 12 ) . However, the overexpression of HGF/SF and c-Met are often detected in multiple types of human cancers and associated with poor prognosis for cancer patients ( 13 ) . In fact, the c-Met signaling pathway has been confirmed to be involved in survival, growth, proliferation, migration, angiogenesis and metastasis of cancer cells during cancer progression ( 14 , 15 ) .

Our previous studies revealed the concomitant down-regulation of both AR and prostate specific membrane antigen (PSMA) expression during long-term androgen-deprivation in an established *in vitro* model ( 16 ) . We further explored whether long androgen-deprived LNCaP cells develop an alternative signaling pathway for growth and proliferation to replace the loss of AR signaling pathway. Our data suggest that long-term androgen deprivation may induce overexpression of c-Met with the downregulation of both AR and PSMA to progress toward a more aggressive, androgen-independent prostate cancer disease state.

## Materials and methods

### Cell lines and reagents

The human prostate cancer cell lines LNCaP and PC-3 were obtained from the American Type Culture Collection (Manassas, VA, USA). The rabbit polyclonal anti-AR antibody (N-20) was obtained from Santa Cruz Biotechnology Inc. (Santa Cruz, CA, USA). The goat anti-rabbit secondary antibody-FITC and the rabbit anti-actin antibody were obtained from Sigma-Aldrich (St. Louis, MO, USA). The mouse monoclonal anti-PSMA antibody 7E11 was graciously provided by Cytogen Corporation (Princeton, NJ, USA). Protein blocking solution was obtained from BioGenex (San Ramon, CA, USA). Rabbit monoclonal anti-c-Met (C-terminus) antibody was obtained from Invitrogen (Grand Island, NY, USA). Rabbit monoclonal anti-c-Met (N-terminus) antibody was obtained from Abcam (Cambridge, MA, USA). Hoechst 33342 were obtained from Invitrogen-Molecular Probes (Carlsbad, CA, USA). Cy5.5-CTT-54.2 was prepared by our lab as described previously ( 17 ) . Halt Protease Inhibitor Cocktail (100X) was purchased from Thermo Fisher Scientific (Rockford, IL, USA). All other chemicals and cell-culture reagents were purchased from Fisher Scientific (Sommerville, NJ, USA) or Sigma-Aldrich.

### Cell culture

LNCaP and PC-3 cells were grown in T-75 flasks with normal growth media [RPMI-1640 containing 10% heat-inactivated fetal calf serum (FBS), 100 units of penicillin and 100 *μ* g/ml streptomycin] in a humidified incubator at 37°C with 5% CO _2_ . Otherwise, for androgen-deprivation growth, cells were cultured with conditioned media [RPMI-1640 containing 10% charcoal-stripped fetal bovine serum, 100 units of penicillin and 100 *μ* g/ml streptomycin]. Confluent cells were detached with a 0.25% trypsin 0.53 mM EDTA solution, harvested, and plated in 2-well slide chambers at a density of 4×10 ^4^ cells/well. Cells were grown for three days before conducting the following experiments.

### Immunofluorescence detection of AR

The LNCaP cells grown under androgen deprivation condition over time (5, 10 and 20 passages) were cultured for 3 days on the slides in the conditioned media. For 2-day androgen-deprivation treatment, LNCaP cells were seeded on slides with normal growth media for 1-day growth, and replaced with conditioned media for another 2-day growth. Normal LNCaP cells and PC-3 cells were used for the AR-positive and AR-negative control, respectively. These cells were seeded on slides with normal growth media for 3 days. Slides with cells grown for 3 days in normal growth media or conditioned media were washed twice in phosphate-buffered saline (PBS), fixed in 4% paraformaldehyde in PBS buffer for 15 min at room temperature, and permeabilized with 0.2% Triton X-100 in PBS buffer for 5 min at room temperature. The permeabilized cells were blocked in block buffer (0.1% Tween-20, 5% goat normal serum in PBS buffer) for 2 h at room temperature and incubated with primary anti-AR antibody (100X diluted in block buffer) overnight at 4°C. After washing, the cells were incubated with a secondary antibody (goat anti-rabbit IgG-FITC, 40X diluted in 1% BSA, PBS buffer) for 2 h at room temperature, counterstained with Hoechst 33342, and mounted in Vectashield ^®^ Mounting Medium (Vector Laboratories Inc., Burlingame, CA, USA) for confocal microscopy.

### Chemofluorescence detection of PSMA

The cells cultured on the 2-well slides were washed twice with warm medium (37°C) A (phosphate-free RPMI-1640 containing 1% FBS), then incubated with 1 ml of Cy5.5-CTT-54.2 (10 *μ* M) in warm medium A for 1 h in a humidified incubator at 37°C and 5% CO _2_ . The above treated cells were washed three times with cold-KRB buffer pH 7.4 (mmol/l: NaCl 154.0, KCl 5.0, CaCl _2_ 2.0, MgCl _2_ 1.0, HEPES 5.0, D-glucose 5.0) and fixed with 4% paraformaldehyde in KRB for 15 min at room temperature. The cellular nuclei were counterstained with Hoechst 33342, and then mounted in Vectashield Mounting medium for confocal microscopy.

### Immunofluorescence detection of c-Met

The cells cultured on the 2-well slides were washed twice with PBS buffer and fixed in 4% paraformaldehyde in PBS buffer for 15 min at room temperature, and permeabilized with pre-chilled methanol for 5 min at −20°C. The fixed cells were blocked in blocking buffer (0.1% Tween-20, 5% goat normal serum in PBS buffer) for 2 h at room temperature and incubated with primary rabbit anti-human c-Met antibody (100X diluted in blocking buffer) overnight at 4°C. After washing, the cells were incubated with a secondary antibody (goat anti-rabbit IgG-FITC, 40X diluted in 1% BSA, PBS buffer) for 2 h at room temperature, counterstained with Hoechst 33342, and mounted in Vectashield Mounting medium (Vector Laboratories Inc.) for confocal microscopy.

### Confocal laser scanning microscopy

Cells were visualized under 40X oil immersion objective using an LSM 510 META Laser Scanning Microscope. Hoechst 33342 was excited with a Diode laser (405 nm), and the emission was collected with a BP420–480 nm filter. AR immunofluorescence (with goat anti-rabbit IgG-FITC) was excited at 488 nm using an Argon Laser, and the emission was collected with an LP505 nm filter. PSMA-targeted imaging with Cy5.5-CTT-54.2 was excited using 633 nm from a HeNe Laser, and the emission collected with an LP 650 nm filter. To reduce interchannel crosstalk, a multi-tracking technique was used, and images were taken at a resolution of 1,024×1,024 pixels. Confocal scanning parameters were set up so that the control cells without treatment did not display background fluorescence. The imaging colors of the fluorescent dyes, Hoechst 33342 and FITC, were defined as blue and green, respectively. When the emission wavelength of the near-infrared fluorescent dye Cy5.5 was beyond visible ranges, fluorescence pseudocolor of Cy5.5 was assigned as red. The pictures were edited by National Institutes of Health (NIH) Image J software ( http://rsb.info.nih.gov/ij ) and Adobe Photoshop CS2.

### Cell morphology

The change in cell morphology was visualized using a compound light microscope (Olympus BH-2, Olympus Optical Co. Ltd., Tokyo, Japan) at ×20 magnification (objective lens numerical aperture =1.25). Digital images were obtained using a digital camera system (Jenoptik ProgRes Camera, C12plus, Jenoptik laser, Optical Systems GmbH, Jena, Germany) mounted on the microscope.

### Whole cell lysate extraction and western blot analysis

The controls: PC-3 and LNCaP cells (cultured in normal growth media) and LNCaP cells under androgen deprivation over time (2 days, 5, 10, or 20 passages) were collected by scraping, washed once in ice-cold PBS, re-suspended in 3-fold cell pellet volumes of lysis buffer (1% NP-40, 20 mM Tris pH 8.0, 137 mM NaCl, 10% glycerol) ( 18 , 19 ) supplemented with 1X Halt Protease Inhibitor Cocktail for 15 min on ice, then transferred to Eppendorf tubes for centrifugation at 10,000 × g for 15 min at 4°C, the supernatant was saved as whole-cell protein extracts. Protein concentrations were determined using Non-Interfering Protein Assay (G-Biosciences, St. Louis, MO, USA). Western blot analysis was performed as described previously with only minor modifications ( 19 , 20 ) . In brief, detergent-soluble proteins (30 *μ* g) were loaded and separated on a NuPAGE™ 4–12% Bis-Tris Gel (Invitrogen, Carlsbad, CA, USA) by electrophoresis for 40 min at a constant 200 V under reducing conditions, and then transferred to a 0.45 *μ* m PVDF Immobilon-P Transfer Membrane (Millipore Corporation, Bedford, MA, USA) at 400 mA for 100 min in a transfer apparatus-Owl Bandit VEP-2 (Owl, Portsmouth, NH, USA) according to the manufacturer’s instructions. Membranes were incubated with primary antibody at corresponding dilution overnight at 4°C and then with horseradish peroxidase conjugated-second antibody for 1 h at room temperature. The immunoreactive bands were visualized using Protein Detector TMB Western Blot kit (KPL, Gaithersburg, MD, USA) following the manufacturer’s instructions.

## Results

### An expression-switch from AR to c-Met induced by androgen deprivation

**Figure 1.**
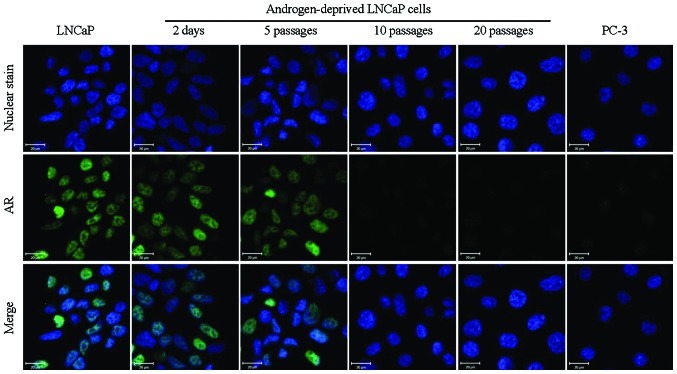
Downregulation of AR expression in LNCaP cells following androgen deprivation treatment. LNCaP cells cultured in normal growth media, served as AR positive controls; strong fluorescence signals for AR (green) were detected throughout the nuclei. Decreased signals for AR were found in LNCaP cells under androgen deprivation conditions for 2 days and 5 passages. No signals for AR were observed for LNCaP cells proliferated under androgen deprivation conditions by 10 passages or in the AR-negative PC-3 cells. Cell nuclei were counterstained with Hoechst 33342 (blue). Cellular imaging was visualized by confocal microscopy; scale bar, 20 *μ* m.

**Figure 2.**
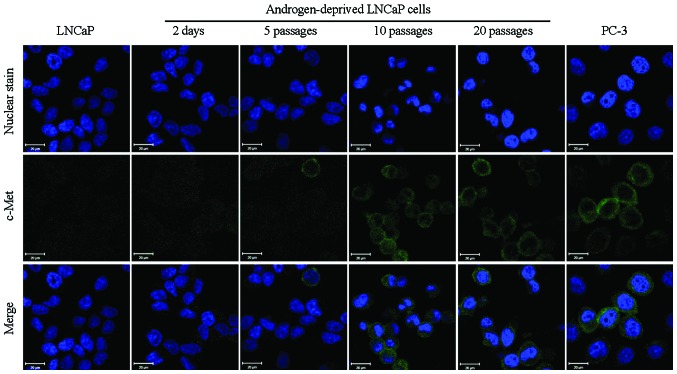
Induced c-Met expression in LNCaP cells following androgen deprivation treatment. PC-3 cells served as c-Met positive controls; fluorescence signals for c-Met (green) were detected on the cell surface. c-Met was found on LNCaP cells proliferated under androgen deprivation conditions by 5 passages and significantly by 10 passages. No signals for c-Met were observed for LNCaP cells cultured in normal growth media. Cell nuclei were counterstained with Hoechst 33342 (blue). Cellular imaging was visualized by confocal microscopy; scale bar, 20 *μ* m.

Cell immunofluorescence imaging clearly demonstrated that downregulation of AR was observed in androgen-deprived LNCaP cells in a time-dependent manner ( Fig. 1 ) and after 10 passages, AR was no longer detectable. In contrast, there were strong signals in the nuclei of normal growth medium-cultured LNCaP cells. As expected, no signal for AR was observed in AR-negative PC-3 cells. However, the immunofluorescence signals for c-Met exhibited a reversed trend of increased expression with time ( Fig. 2 ) with considerable expression of c-Met by 10 passages in androgen deprived growth medium. These results suggest that the switch from AR to c-Met expression is an adaptable response by the LNCaP cell line to prolonged androgen deprivation. Although these results were not consistent with a previous report which demonstrated that the administration of androgens resulted in increased AR mRNA levels in LNCaP cells ( 21 ) , our data are strongly supported by the analysis of clinical prostate cancer specimens ( 22 , 23 ) .

### Loss of PSMA during prolonged androgen deprivation

**Figure 3.**
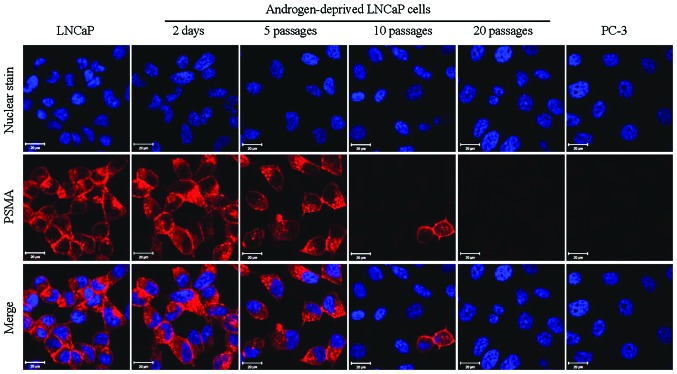
Loss of PSMA expression in androgen-deprived LNCaP cells following androgen deprivation treatment. LNCaP cells cultured in normal growth media, served as PSMA-positive controls; fluorescence signals for PSMA (red) were detected on the cell surface. Decreased signals for PSMA were found in LNCaP cells proliferated under androgen deprivation conditions by 10 passages. No signals for PSMA were observed for either LNCaP cells proliferated under androgen deprivation conditions by 20 passages or PSMA-negative PC-3 cells. Cell nuclei were counterstained with Hoechst 33342 (blue). Cellular imaging was visualized by confocal microscopy; scale bar, 20 *μ* m.

Cy5.5-CTT-54.2, a specific PSMA fluorescent inhibitor (IC _50_ = 0.55 nM) was prepared and evaluated for PSMA-targeted fluorescence imaging of LNCaP cells in a previous investigation from our group ( 17 ) . In the present study, Cy5.5-CTT-54.2 was employed to detect the change of functional PSMA on the cell surface of androgen-deprived LNCaP cells by fluorescence imaging. Consistent with the results for AR immunofluorescence study above, the considerable cell labeling by Cy5.5-CTT-54.2 observed for LNCaP cells either cultured in normal growth media or proliferated under androgen deprivation conditions for up to 5 passages. However, PSMA expression was significantly reduced by 10 passages in LNCaP cells ( Fig. 3 ) and by 20 passages PSMA expression was similar to that of PSMA-negative PC-3 cells.

### Changes of cell morphology

**Figure 4.**
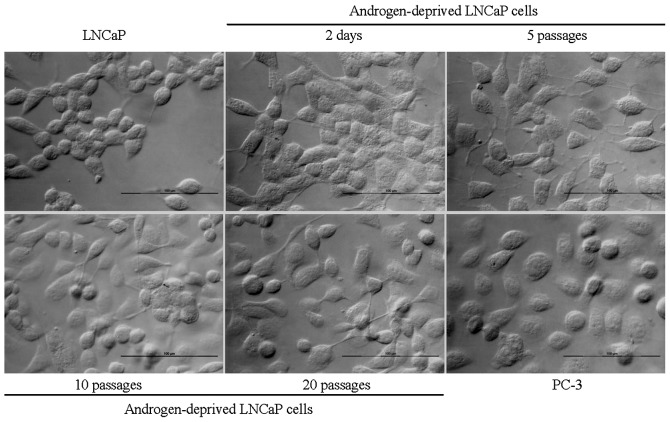
Induced morphological changes in LNCaP cells following androgen deprivation treatment. PC-3 cells and LNCaP cells cultured in normal growth medium serve as references. The loss of tight cell-cell contact and a more scattered growth pattern were found in LNCaP cells proliferated under androgen deprivation conditions over time. Cellular imaging was visualized by a light-contrast microscope; scale bar, 100 *μ* m.

The main morphological change of androgen-deprived LNCaP is the loss of cell to cell tight contact and changed to scattered growth. The change is clearly correlated with the protein expression switch from AR to c-Met, which indicated that cells became more aggressive ( Fig. 4 ).

### Western blot analysis

**Figure 5.**
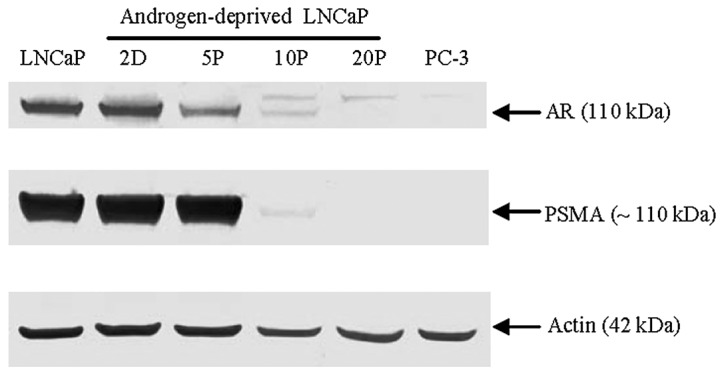
Western blot analysis detection of AR and PSMA expression. Compared to LNCaP cells cultured in normal growth medium, PSMA expression is significantly reduced in LNCaP cells proliferated under androgen deprivation conditions by 10 passages. No signals for PSMA were observed for either LNCaP cells proliferated under androgen deprivation conditions by 20 passages or PSMA-negative PC-3 cells. Actin expression was detected to serve as a protein loading control.

**Figure 6.**
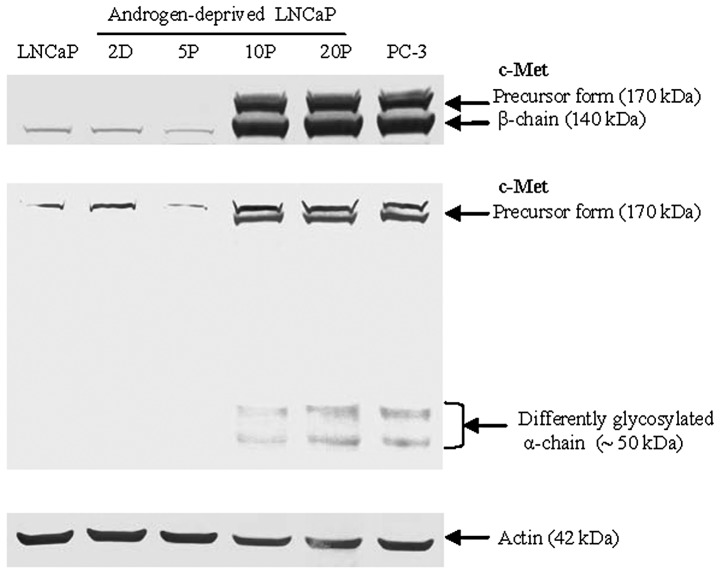
Western blot analysis detection of c-Met expression. Strong c-Met expression was detected in LNCaP cells proliferated under androgen deprivation conditions by 10 passages (10P) and 20 passages (20P). PC-3 cells served as c-Met positive controls. Actin expression was detected to serve as a protein loading control.

Western blot analysis further confirmed that the total amount of AR and PSMA expression decreased in LNCaP cells over the course of androgen deprivation conditions with a dramatic loss by 10 passages and absence after 20 passages; actin served as a protein loading control ( Fig. 5 ). In contrast, an inducible upregulation of c-Met expression was strongly detected by 10 passages of LNCaP cells proliferated under androgen deprivation conditions using two different epitope antigen-binding antibodies ( Fig. 6 ). These data suggest that downregulation of AR and the expression of c-Met in the model LNCaP cell line is dependent on androgen levels and the duration of androgen deprivation during cell culture. A cell signaling pathway switch from AR to c-Met in prostate cancer may originate from the selective pressure of long-term androgen deprivation.

## Discussion

Although CRPC cells may retain active AR and be sensitive to anti-AR therapies, our data suggest that long-term androgen-depletion may induce a signaling pathway switch from AR to c-Met, which may lead to a diagnostically and therapeutically elusive androgen-independent disease state. However, this switch to c-Met dependence for proliferation may help to define new therapeutic strategies tailored specifically against CRPC by targeting the c-Met signaling pathway through the use of a c-Met inhibitor, some of which are currently being investigated clinically ( 24 ) .

The HGF/c-Met signaling pathway plays a key role in tumorigenesis and tumor progression. Overexpression of c-Met has been frequently found in advanced, metastatic, and castration-resistant prostate cancers ( 25 ) . An inverse correlation between the expression of AR and c-Met has been observed in prostate epithelium and prostate cancer cell lines ( 9 , 25 ) , implying that these receptors may represent the state of the androgen switch in prostate cancer cells. This is also supported by findings revealing that AR negatively regulates c-Met expression in cell models ( 12 ) . In addition, high expression of c-Met in prostate cancer may also be correlated to bone metastasis ( 26 ) .

In summary, our present data support the concept that long-term androgen deprivation promotes a signaling pathway switch from AR to c-Met leading to the development of an aggressive phenotype of prostate cancer. It is expected that early detection of this signaling switch may provide critical information in treatment planning for prostate cancer patients with recurrent, metastatic and castration-resistant prostate cancer.
